# Attitudes toward Grandparental Involvement in Hong Kong: A Trend Analysis

**DOI:** 10.3390/ijerph19169858

**Published:** 2022-08-10

**Authors:** Mengtong Chen, Qiqi Chen, Camilla Kin Ming Lo, Susan J. Kelley, Ko Ling Chan, Patrick Ip

**Affiliations:** 1 Department of Social Work, The Chinese University of Hong Kong, Ma Liu Shui, Hong Kong, China; 2 Department of Social Work, School of Sociology and Anthropology, Xiamen University, Xiamen 361005, China; 3 Department of Applied Social Sciences, The Hong Kong Polytechnic University, Hung Hom, Hong Kong, China; 4 Byrdine F. Lewis College of Nursing and Health Professions, Georgia State University, Atlanta, GA 30303, USA; 5 Department of Paediatrics and Adolescent Medicine, LKS Faculty of Medicine, The University of Hong Kong, Pokfulam, Hong Kong, China

**Keywords:** grandparent, family relations, family structure, Hong Kong

## Abstract

This article examines individuals’ attitudes toward the involvement of grandparents in family issues in Hong Kong. While existing studies have largely focused on the nature and types of grandparents’ involvement in childcare, it is worth conducting a quantitative investigation of the attitudes in the general population about grandparental involvement. Drawing on the 2011, 2013, 2015, and 2017 waves of the Family Surveys, the study examined the trend in attitudes toward grandparental involvement with 8932 HK residents. Multivariate linear regression analyses were performed to assess individual and family relationship factors associated with the attitudes toward grandparental involvement. Results show that although most people held positive attitudes toward grandparental involvement, there was a significant drop in the agreement with grandparental involvement in 2017 across all age groups. The findings imply that intergenerational support tends to be weakened in HK in recent years. Involving grandparents in family issues in HK was more likely to be need-driven rather than value-driven, as parent respondents had relatively more positive attitudes toward grandparental involvement compared with non-parents. Positive family and intergenerational relationships were significantly associated with the positive attitudes toward grandparental involvement. Policymakers and service providers should recognize the changes in people’s attitudes toward family lives and provide appropriate support such as family counselling, (grand)parenting programs and childcare support to promote the wellbeing of families and older adults.

## 1. Introduction

With improved health status of older adults and increased longevity, grandparenthood constitutes a significant part of one’s aging process [1]. Although grandparents tend to apply the norm of noninterference in intergenerational relationships today, especially in Western societies, many of them play an important role in providing childcare to families [2,3]. Grandparents are common resources of informal social support in both Western and Eastern cultural contexts in response to the childcare needs of adult children due to different family conditions. There appear to be two distinct groups of grandparent caregivers in providing childcare, i.e., custodial grandparents and supportive grandparents, although there is heterogeneity in the grandparenting role in terms of the different demographic characteristics, geographical proximity, and care regimes and contexts [4].

Custodial grandparents refer to those who are providing primary care for grandchildren on a full-time basis in the absence of the grandchildren’s biological parents [5,6]. The increasing prevalence of custodial grandparenting has attracted great attention in the United States [7]. In 2019, approximately 1.1 million grandparents in the US provided childcare for coresident grandchildren [8]. It is estimated that 8.4% of children live in grandparent-headed households [9], mainly due to parents’ inability to care for children, e.g., drug abuse, incarceration, domestic violence, economic instability, military deployment, and mental illness [5]. A disproportionate number of African American grandparents serve important roles as custodial grandparents, also partly due to the cultural diversity of grandparenthood [10]. Compared to Caucasian grandparents who define family as nuclear in nature, African American grandparents may view family as an “ongoing entity” [11,12]. Supportive grandparents refer to the older adults who engage in childcare as a supplement to parental childcare [13]. According to the Survey of Health, Ageing and Retirement in Europe (SHARE), grandparents were regarded as regular caregivers if they provided grandchild care almost daily or for at least 15 h per week [2]. Providing regular or occasional childcare support can help parents of young children reconcile their work and family roles, especially in countries without strong formal childcare systems [14,15]. Generally speaking, despite a higher prevalence of individualistic values, grandparents provide different intensities of care to grandchildren in Western countries [15].

Grandparents hold a unique role in the East Asian countries influenced by Confucianism. Confucianism is one of the fundamental philosophies in the East Asian cultural sphere and has long influenced the conceptualizations of family structure and the perceptions of the role of grandparents of East Asian people. Confucianism highlights an individual’s virtues and emphasizes achieving harmony in the family [16]. Filial piety is a virtue that requires showing respect for grandparents, who are regarded as heads of the households and role models for the younger generation. Benevolence is a virtue that requires caring about people. As such, benevolence for children is a moral obligation for family members and kinships. Therefore, historically and culturally, intergenerational coresidence is very common in East Asian countries. Intergenerational coresidence is still a prevalent living arrangement today. However, there has been a significant shift in family structure from the extended family to the nuclear family in contemporary East Asian societies. In South Korea, about 28% of individuals aged 65 years and above coresided with their children [17]. In addition, 37.2% of South Korean families with preschool children receive intensive childcare support from grandparents [18]. In China, multigenerational households accounted for 16.7% of all households [19]. Almost two-thirds of Chinese grandparents are involved in childcare [20]. In addition to the embedded Confucian perspectives, increased labor force participation of women and the lack of affordable formal childcare also contribute to grandparents’ engagement in childcare. Grandparents’ involvement in Chinese families also varies from occasional involvement in childcare to custodial grandparenting. The increased modernization in China has caused parents to migrate to cities and leave behind their families in search of employment. Thus, a large number of rural grandparents have to raise their grandchildren on a full-time basis.

While custodial grandparenting tends to imply the dysfunctioning of a nuclear family consisting of parents and children, it also indicates the vital role that grandparents play in preserving family functioning to provide a healthy environment for child growth. In the global trends in demographic change and family transformations, grandparents are a critical resource for families to build resilience against adversities and stress [3]. In Chinese migrant families, grandparents serving as primary caregivers of children can compensate for the absence of parents to some extent [21]. Supportive grandparents also play an essential role in promoting optimal family functioning, as their assistance allows the mothers to participate in the labor market [15]. Their involvement also improves grandchildren’s cognitive and social adjustment and quality of life [22,23].

### 1.1. Attitudes toward Grandparental Involvement: The Family Systems and Life Course Perspectives

The family systems perspective provides a useful conceptual framework to understand intergenerational solidarity, as it focuses on the interdependence among the subsystems (e.g., marital, parental, and sibling subsystems) that comprise families. This perspective suggests that all dyadic family relationships are embedded within the whole family system and that each relationship influences and is influenced by other relationships [24]. Particularly, the emotional contact between multigenerational family members can enhance family functioning [25]. The importance of multigenerational bonds is increasingly recognized in the literature, as existing studies on grandparents’ involvement in family issues have largely focused on the nature and types of grandparents’ involvement in childcare [5]. Within the subsystems of grandparents and other family members, the different attitudes toward grandparental involvement may trigger changes in the intergenerational relationship and affect family functioning.

The life course perspective underlines the importance of different temporal and social contexts on the wellbeing of individuals and generations [26]. It emphasizes that individual development is characterized by diverse trajectories over time, with all life stages intricately intertwined. Becoming a parent is an important stage for an individual’s life course, which may predict the attitudes toward grandparental involvement. Furthermore, the attitudes toward grandparental involvement may also affect such transition in the life course. In addition to the ontogenic time that describes the life course of an individual, generational time which refers to the position of an individual within the family (i.e., younger, middle, or older generations) may also affect the attitudes toward grandparental involvement.

A few studies have examined the perceptions of grandparents who engaged in childcare and parental perceptions of the importance of grandparents in childcare. A study based on New Zealand samples shows that though the grandparents agreed with their roles as knowledge holders to pass on cultural tradition and values to grandchildren, they also felt that providing childcare deprived them of time [27]. Such ambivalence was also observed among Spanish grandparents. Despite some reporting being burdened by childcare, the grandparents generally recognized the importance of their role in the socialization of grandchildren [28]. Mothers of children with disabilities explicitly expressed that grandparents served as an important source of instrumental and emotional support and provided more assistance compared to other informal social supports [29,30]. Perceived respect from younger generations would increase the generative concern of older adults, which may increase the levels of psychological wellbeing in old age [31]. Older women who provided childcare and financial support to their adult children had higher levels of life satisfaction if they perceived their children as being devoted to them [32]. Therefore, it is important that grandparent support for adult children is recognized and that they receive intergenerational exchanges of emotional, instrumental, or financial support.

In trying to understand the attitudes toward grandparental involvement in family issues, most studies were qualitative explorations of the experiences of caregiving grandparents and mothers. Focusing on respondents who provided or received grandparental support can result in selection bias and hamper the generalization of findings. As attitudes toward grandparental involvement could be an important indicator of people’s perception of family functioning, it is worth conducting a quantitative investigation of the attitudes in the general population about grandparental involvement. Specifically, as attitudes toward grandparental involvement have implications on family transitions such as childbirth [15], there can be discrepancies between parents and non-parents. In East Asian countries where grandparents have historically engaged in family lives, an emerging cohort of “new” grandparents demand freedom without feeling constrained by family obligations in the recent decade [3]. The attitudes of younger generations toward grandparental involvement may also change in the face of broad social, economic, and political transformation. It is meaningful to know the trends of attitudes toward grandparents’ involvement over time among the general population and the discrepancy in the perceptions between age groups.

### 1.2. Family Structure Changes and Grandparenthood in Hong Kong

Hong Kong is a unique city lying in South China where East meets West. Despite Western cultural influences, the Chinese cultural values about the importance of family, filial piety, and harmony also influence all aspects of family lives [33]. However, Hong Kong has undergone rapid demographic and family transformations over the past three decades. Though the notion of filial piety as a cultural norm still runs deep in Hong Kong, the norm is undergoing modification to suit one’s own experiences and circumstances [34,35].

Hong Kong is an aging society with very low fertility [36]. The changes in population structure have led to changes in family structure. Though the nuclear family has become the predominant form of domestic households since the 1970s, its proportion has dropped to only 36.7%, according to the 2016 Hong Kong Population By-census; and in comparison, 15.5% of households were composed of couples only [37,38]. The three-generation extended family represented only 3.7% of households in Hong Kong [37]. In addition, Hong Kong is one of the most densely populated places in the world, as such small or even nano apartments have proliferated in modern housing design in recent decades. Only 16.5% of public rental houses in Hong Kong were larger than 40 m^2^ [39]. Due to the limited living space, it has become difficult for different generations to live together.

In recent decades, multigenerational coresidence is not common or deemed preferable in Hong Kong. While the traditional filial piety value focuses heavily on the obligations of children to parents, it has weakened in the face of economic and political transformation. The filial relationship is sometimes manifested as the simple financial support from adult children, as most adult children offer part of their incomes to their parents to repay them for the love and support they received throughout childhood and to show their care and respect toward parents [34]. Nonetheless, intergenerational relationships remain essential, as grandparents are still a significant source of informal support to families and actively engage in different aspects of family lives. According to the 2017 Hong Kong Family Survey, approximately one in three parents in Hong Kong reported that their parents had helped them raise children; in addition, over half of parents agreed that they would help raise grandchildren in the future [40]. In another survey on families with children aged 6–48 months, about 25% of the families received intensive childcare from grandparents, who were the principal child caregivers during the daytime, and 30% of the infants or toddlers lived with their grandparents for five or more days a week [41]. Grandparental involvement is a source of informal social support for the family, supplementing formal social service support, which is always insufficient in Hong Kong [42].

### 1.3. Research Objectives

With the decline in multigenerational households and shrinking family size, attitudes toward the importance of grandparental involvement may change over time in Hong Kong. In the current study, we seek to describe the attitudes toward grandparental involvement in family issues, in particular childcare, and in part, to shed light on family functioning in modern Hong Kong society. The first objective is to study the general trend in people’s attitudes toward grandparental involvement as an essential step toward understanding family functioning. The second objective is to examine the attitudes toward grandparental involvement of both parents and non-parents of different age groups. By identifying the groups of families who perceive grandparental involvement as necessary, the article will shed light on the in-depth understanding of family structure change and family functioning in Hong Kong families. The third objective is to investigate individual and family level factors that are associated with people’s attitudes toward grandparental involvement. We mainly investigated the role of intergenerational solidarity, i.e., social cohesion between generations [43]. This study is a preliminary quantitative exploration of people’s attitudes toward grandparental involvement based on data from the four waves of Family Surveys in Hong Kong.

## 2. Methodology

### 2.1. Research Design and Sample

This study used secondary quantitative data from Family Surveys commissioned by the Home Affairs Bureau of the Government of the Hong Kong SAR, China. Aiming to track the changes and development among Hong Kong families, the surveys were carried out on a biennial basis from 2011 to 2017. A two-stage stratified sample design was adopted to randomly sample households in Hong Kong. In the first stage, a list of quarters was randomly selected based on the lists of Register of Quarters and Register of Segments obtained from the Census & Statistics Department (C&SD). In the second stage, a family member aged 15 years or above residing in Hong Kong in each household was randomly selected for an interview based on the last birthday principle. The average response rate of the surveys was 63.5%. The population trend surveys were cross-sectional. For each of the first three waves of the survey, the sample size was 2000. Moreover, the sample size at the fourth wave was 2932. The total sample for the study comprised 8932 persons.

### 2.2. Measurements

#### 2.2.1. Attitudes toward Grandparental Involvement

Individuals’ attitudes toward “the involvement of grandparents in family issues” were examined as a dimension of the attitudes concerning the importance of family in the Family Surveys. The index regarding attitudes toward grandparental involvement consisted of four items (Cronbach’s α > 0.7): (1) many parents today appreciate the help that grandparents give, (2) people today place enough value on the part grandparents play in family life, (3) in most families, grandparents should be closely involved in deciding how their grandchildren are brought up, and (4) with so many working mothers, families increasingly need grandparents’ help. Respondents indicated their level of agreement on a five-point Likert scale (ranging from 1 = strongly disagree to 5 = strongly agree). A higher score indicates more positive attitudes toward grandparental involvement in family issues.

#### 2.2.2. Correlates of Attitudes toward Grandparental Involvement

Demographic characteristics, socioeconomic status (SES), having children or not, intergenerational solidarity, satisfaction with family relationships, and satisfaction with life were studied as correlates. Demographic characteristics included respondents’ age, gender, and marital status. SES included respondents’ educational level, whether they were economically active or not, and whether they were new arrivals to Hong Kong.

In the current study, intergenerational solidarity included affectual solidarity and associational solidarity, which were measured by closeness to family members and communications with family members, respectively. Respondents were asked about how close they felt to family members and inter-generations, rating on a four-point Likert scale (ranging from 1 = not close at all to 4 = very close). They were also asked about how frequently they talked about personal issues with family members and inter-generations, rating on a four-point Likert scale (ranging from 1 = almost never to 4 = frequently). Regarding the satisfaction with family relationships and family lives, respondents were asked to indicate their level of satisfaction by two items on a five-point Likert scale (ranging from 1 = very dissatisfied to 5 = very satisfied).

### 2.3. Statistical Analysis

First, descriptive analyses were conducted to summarize respondents’ demographic characteristics and to examine the trend of individuals’ attitudes toward the involvement of grandparents in family issues. The attitudes toward grandparental involvement in different age groups among parents and non-parents were also examined. Second, a multivariate regression analysis was performed to estimate the associations between life satisfaction and each of the family relationship variables and the attitudes toward grandparental involvement with the wave of survey. All of the demographic, SES and family structure variables were entered in the model. The four-wave samples were combined into an aggregate sample in the regression analyses.

## 3. Results

### 3.1. Characteristics of Respondents

The respondents’ characteristics in the four waves of the survey are shown in Table 1. More participants were in the older age groups across the four waves. Slightly more than half of the participants were married or cohabiting with a partner and about one in four were never married. Approximately 70% of the respondents had a secondary educational level or above. Less than half of the respondents were economically active. Over 60% of respondents were parents. However, fewer than 50% of the respondents were married and cohabiting with children. Only a small proportion of respondents were new immigrants, or “new arrivals”, who had stayed in Hong Kong less than 7 years at the time of the survey.

### 3.2. Trend of Attitudes toward Grandparental Involvement

Table 2 shows respondents’ attitudes toward grandparents’ involvement in family issues. In general, more respondents agreed with the statements about grandparental involvement across four waves from 2011 to 2017, but a significant drop was observed in the fourth wave. In the fourth wave, fewer than 50% of the respondents showed positive views on grandparental involvement, and over one-third showed neutral attitudes. Significant increases in negative views on grandparental involvement were also observed. The lowest scores were observed on the item “in most families, grandparents should be closely involved in deciding how their grandchildren are brought up”, as approximately one third of the respondents disagreed with this statement in 2017. While about 50% of the respondents agreed with the item “people today place enough value on the part grandparents play in family life” in the first three waves from 2011 to 2015, the percentage dropped to 40% in 2017.

### 3.3. Attitudes toward Grandparental Involvement by Parental Role and Age Group

As shown in Table 3, there were also significant drops in the positive attitudes toward grandparental involvement across all age groups in wave four, though the rates of decrease were slightly slower among the age group 55 years and above. Compared to non-parents, the parent respondents reported higher levels of agreement with the statements about grandparental involvement across almost all age groups and waves. The respondents in the 35–54 age group reported the lowest levels of agreement with almost all the items. Regarding the item about whether to involve grandparents in decision making about childrearing, the 35–54 age group also reported a very low level of agreement. It should be noted that although there were some very high levels of agreement among parent respondents between 15 and 24 years in the first and second waves, it may be due to the smaller sample size of this sub-group.

### 3.4. Factors Associated with Attitudes toward Grandparental Involvement

As shown in Table 4, Model 1 shows that respondents in Wave 2011 (Beta = 0.246, *p* < 0.001), Wave 2013 (Beta = 0.245, *p* < 0.001), and Wave 2015 (Beta = 0.252, *p* < 0.001) demonstrated more positive attitudes toward grandparental involvement compared to participants in Wave 2017. Model 2 shows that the variables statistically significantly associated with the attitudes toward grandparental involvement also included younger (Beta = 0.086, *p* < 0.01) and middle (Beta = −0.071, *p* < 0.001) age groups, male gender (Beta = 0.034, *p* < 0.05), and having children (Beta = 0.126, *p* < 0.001). In Model 3, when family relationship variables were added to the regression model, the effect of younger age became insignificant, whereas being economically active (Beta = 0.037, *p* < 0.05), being a new arrival in Hong Kong (Beta = 0.092, *p* < 0.05), having a higher level of satisfaction with relationships between family members and inter-generations (Beta = 0.100, *p* < 0.001), having a higher level of communications with family members and inter-generations (Beta = 0.034, *p* < 0.001), and having a higher level of satisfaction with family life (Beta = 0.041, *p* < 0.001) were significantly associated with respondents’ attitudes toward grandparental involvement.

## 4. Discussion

Although some qualitative studies have explored the attitudes toward grandparental involvement, it remains unclear about the universal perceptions on the involvement of grandparents in family issues. Hong Kong has a long tradition of intergenerational care transfer, notwithstanding that the society has come under heavy Western influence. However, due to a decreased fertility rate, shrinking family size, and a decreased proportion of nuclear families in Hong Kong, the importance of family is diminishing and the role of grandparents in childcare seems to be weakened. Based on an analysis of secondary data from the four waves of Family Surveys in Hong Kong, the current study reveals that though a larger number of people held positive attitudes toward grandparental involvement, their attitudes exhibited a decreasing trend in the fourth wave. In the year 2017, over one-third of the respondents reported only a neutral attitude toward grandparental involvement. The findings imply that family functioning in terms of intergenerational support tends to be weakened along with the demographic, economic, and sociopolitical changes. This result also echoes the increase in family disorganization, which is significantly associated with the unacceptable level of poverty in Hong Kong [44]. Grandparents’ participation in the decision making of the childrearing process rated the lowest amongst the respondents. In traditional Chinese families, grandparents usually cling to a hierarchical parenting style, believing that they should guide the decision making about childrearing [45]. However, the findings show that the respondents were relatively cautious about the power relations between parents and grandparents in the childrearing process. Grandparents are expected to play an assistant role in childcare and adhere to the norm of non-interference. Furthermore, grandparents may be perceived as lacking knowledge in modern nurturing styles, which devalues their experiences in childrearing [46].

The current study also reveals that the attitudes toward grandparental involvement varied across different age groups and between parents and non-parents. Parent respondents had relatively more positive attitudes toward grandparental involvement, and non-parents reported lower levels of agreement with grandparental involvement. Therefore, involving grandparents in family issues in Hong Kong was more likely to be need-driven rather than value-driven, as there is a pressing need for grandparents’ childcare support in dual-worker families and single-parent families [23]. Research has suggested that grandparental propensity to provide childcare has a positive effect on adult children’s intention to have children [15]. Non-parents in the surveys generally held fewer positive attitudes about grandparental involvement, which implies the decreasing family self-reliance ideology on childcare, thus the low fertility rate in Hong Kong could even prevail. While the traditional nuclear families appreciated the importance of grandparental involvement, the Confucian ideal of cohesiveness could still be significantly challenged due to the decreasing fertility rate and marriage rate in Hong Kong.

Younger people between the ages of 15 and 24 were more likely to value the importance of grandparental involvement. Research has shown that grandparents play an important role in child development, as their involvement can contribute to the cognitive and social adjustment of adolescents and emerging adults [23]. Thus, younger people can maintain an intimate relationship in the interaction with their benevolent grandparents. In comparison, middle-aged adults between the age 35 and 54 reported the lowest levels of agreement with grandparental involvement, especially when asked about whether involving grandparents in decision making about childrearing. The findings show that compared to the close relationship with grandparents maintained by the younger generation, the relationship between middle-aged adults and the older generation might be more complex, which may involve power struggles and even conflict in childrearing [47,48]. The findings also imply a modification of filial piety and the parent–child relationship in modern Hong Kong, where grandparents do not gain absolute obedience from their adult children. As adults 55 years old and above showed more positive attitudes toward grandparental involvement, there could be tensions within families due to the different expectations. Middle-aged adults who did not have children between the ages of 35 and 54 may hold more positive attitudes toward individualism, thus not showing enthusiasm for grandparental involvement.

The findings suggest that both individual and family context factors were associated with respondents’ attitudes toward grandparental involvement. It is not surprising to learn that the most significant correlate was whether the respondent was a parent. Respondents who were male generally held more positive attitudes toward grandparental involvement. While fathers are increasingly involved in childcare in Chinese nuclear families, gendered patterns of childcare and housework remain salient as an influence of the traditional patriarchal ideology [49]. When support from grandparents is available, fathers may totally withdraw from childcare [50]. Compared to grandmothers, who usually take on more childcare responsibilities, grandfathers are often only involved in companionship and sharing family values [51]. New arrivals held more positive attitudes toward grandparental involvement, as they were usually more adherent to traditional family values in Chinese culture. The results also indicate that intergenerational solidarity that manifests in the high levels of emotional bonding between family members and across generations was significantly associated with positive attitudes toward grandparental involvement. As involving grandparents in childcare as supplementary caregivers can be an indicator of family functioning, it is therefore associated with higher levels of satisfaction with family relationships and family life.

### 4.1. Limitations and Implications for Research

Findings of this study need to be interpreted with the following limitations. First, the population surveys did not provide information on individual identifiers; hence we could only study the trend in change at population level but were not able to track the changes over time in individuals. In the current study, though the trend of people’s attitudes toward grandparental involvement suggests that the traditional reliance on grandparents as childcare providers may be fading with time in Hong Kong, we cannot draw conclusions about the causal relationships between life trajectories and change in attitudes toward grandparental involvement, or a wide range of family issues. In future family surveys, a longitudinal study is recommended to track individuals’ attitudes toward family issues over time and elucidate the factors that influence attitudes. In addition, the impact of the changes in attitudes on family wellbeing remains to be investigated in the future. Second, the measurement of attitudes deserves close attention. Though the self-report measurement showed adequate reliability in the current study, there is also a possibility that the respondents’ answers were influenced by social desirability. There could be a discrepancy in respondents’ expressed attitudes and tested attitudes. Third, it is still unclear how the grandparent status among the respondents was associated with their attitudes toward grandparental involvement, as the variable was not examined in the Family Surveys. Fourth, only one family member aged 15 or above in each household was interviewed, therefore, we cannot conclude whether there was consensus about the involvement of grandparents in family issues among family members of different generations. It would be beneficial for future studies to include more family members in in-depth interviews to understand the notion of filial piety or reciprocity in the intergenerational relationships in contemporary society.

### 4.2. Implications for Policy and Practices

Family values and attitudes have become more heterogeneous in Hong Kong [52]. This study shows that the positive attitudes toward grandparental involvement in family issues have decreased in general. While it is not surprising to learn that parents generally showed more positive views on grandparental involvement compared to non-parents, the role of grandparents could be challenged due to the family structure and demographic changes in Hong Kong. Though the support from grandparents is essential for family wellbeing, the role of intergenerational support in childcare seems to reduce gradually, which was observed by people in different age groups. It is important for policy makers and service providers to understand the changes in people’s perceptions regarding grandparental involvement and to deliver appropriate support for families.

First, grandparental involvement can be meaningful to maintain the considerable stability of the affectual relationships between family members and benefit the wellbeing of older adults to some extent [32]. By investigating the attitudes toward grandparental involvement, we may estimate how likely families in Hong Kong are to obtain support from grandparents. In order for grandparents to make a positive impact on family wellbeing, policy makers and service providers can encourage grandparents to be actively involved in family issues. Second, support for families and grandparents is also necessary in case of ambivalence or conflict between family members. For example, it was found that parenting stress was higher among families with grandparental involvement in childcare compared to those who employed domestic helpers [53]. Therefore, both parents and grandparents would benefit from (grand)parenting education and counselling services related to intergenerational relationships. Third, considering the decreased reliance on grandparents to provide childcare support, formal childcare services would be increasingly demanded. It is important for the government to enact more family-friendly policies and provide adequate childcare services. Fourth, during COVID-19, grandparents can be more involved in childcare as public childcare provision and schools are shut but also experience more grandparenting stress. More interventions should be developed to increase the psychological wellbeing of grandparents.

## 5. Conclusions

This study is the first to investigate the attitudes toward grandparental involvement in family issues among the general population. The study found that, on the one hand, Hong Kong people held positive attitudes toward grandparental involvement. However, on the other hand, a significant drop in positive attitudes was observed in the most recent family survey. There might be a bidirectional association between the change in having children and families’ attitudes toward grandparental involvement. It is important that policymakers and service providers recognize the influences of changes in people’s attitudes toward grandparental involvement on family wellbeing and social welfare and provide appropriate family support.

## Figures and Tables

**Table 1:** Characteristics of Respondents (%).

	2011 (*n* = 2000)	2013 (*n* = 2000)	2015 (*n* = 2000)	2017 (*n* = 2932)
Age				
15–24	13.5	11.3	9.9	11.3
25–34	11.2	10.1	9.2	10.5
35–54	34.3	32.6	32.9	30.1
55 or above	41.1	46.1	48.1	48.1
Marital status				
Never Married	27.4	24.1	25.9	28.1
Married/cohabiting	55.4	56.7	55.8	53.7
Divorced/separated/widowed	17.2	19.2	18.3	18.2
Economic status				
Economically active	42.5	39.8	41.5	44.6
Economically inactive	57.5	60.2	58.5	55.4
Educational level				
Primary or lower education	31.6	33.9	32.6	29.0
Secondary educational level	53.0	50.9	53.2	51.4
Post-secondary education or above	15.4	15.2	14.2	19.5
Having children	62.8	68.5	65.1	65.7
Family structure				
Married/cohabiting with child	47.7	50.8	49.7	47.4
Married/cohabiting without child	7.7	5.9	6.1	6.3
Never married	27.4	24.1	25.9	28.1
Divorced/separated	10.7	12.8	10.7	6.7
Widowed	6.5	6.4	7.6	11.4
Length of residence in Hong Kong				
New arrivals (Less than 7 years)	4.2	3.7	3.1	0.4
Not new arrivals (More than 7 years)	95.8	96.3	96.9	99.6

**Table 2:** Attitudes toward the Involvement of Grandparents in Family Issues (%).

	2011	2013	2015	2017
(1) Many parents today appreciate the help that grandparents give.				
Agree/Strongly Agree	60.2	64.8	60.5	48.9
Neutral	29.9	20.5	29.2	34.4
Disagree/Strongly disagree	9.8	14.7	10.4	16.7
(2) People today place enough value on the part grandparents play in family life.				
Agree/Strongly Agree	51.8	58.8	51.0	40.5
Neutral	35.6	24.7	36.6	36.3
Disagree/Strongly disagree	12.6	16.5	12.3	23.1
(3) In most families, grandparents should be closely involved in deciding how their grandchildren are brought up.				
Agree/Strongly Agree	44.5	49.8	42.0	31.4
Neutral	38.9	26.0	41.9	40.0
Disagree/Strongly disagree	16.6	24.1	16.2	28.7
(4) With so many working mothers, families increasingly need grandparents’ help.				
Agree/Strongly Agree	60.1	62.7	61.1	43.8
Neutral	30.0	23.2	30.3	35.5
Disagree/Strongly disagree	10.0	14.1	8.5	20.7

**Table 3:** Agreement (%) on Attitudes Toward the Involvement of Grandparents by Parental Role and Age Group.

	2011				2013				2015				2017			
	15–24	25–34	35–54	55 or above	15–24	25–34	35–54	55 or above	15–24	25–34	35–54	55 or above	15–24	25–34	35–54	55 or above
(1) Many parents today appreciate the help that grandparents give.																
Parent	100.0	64.2	60.5	64.1	100.0	69.2	67.4	66.1	-	72.0	62.6	60.9	51.4	50.6	46.4	54.2
Non-parent	60.8	59.4	47.5	53.8	63.6	66.7	55.6	52.6	60.7	60.3	60.0	50.3	48.6	40.1	44.6	40.6
*p* value	0.016				0.169				0.416				<0.001			
(2) People today place enough value on the part grandparents play in family life.																
Parent	100.0	52.2	51.5	55.5	50.0	66.2	63.2	59.2	-	62.0	53.2	50.7	43.2	45.6	38.0	45.4
Non-parent	53.0	53.8	37.0	48.5	57.7	58.5	47.0	51.6	54.4	52.3	47.5	42.4	38.6	36.9	32.7	33.8
*p* value	0.052				0.411				0.222				0.001			
(3) In most families, grandparents should be closely involved in deciding how their grandchildren are brought up.																
Parent	100.0	43.3	43.8	48.2	50.0	38.5	48.6	56.3	-	53.1	45.8	42.4	29.7	29.1	28.3	37.9
Non-parent	44.6	42.9	31.5	47.7	44.6	44.4	41.9	42.1	38.3	36.4	43.0	34.1	27.8	24.8	25.1	23.9
*p* value	0.002				0.001				<0.001				<0.001			
(4) With so many working mothers, families increasingly need grandparents’ help.																
Parent	100.0	61.2	60.8	67.2	50.0	67.7	66.2	66.1	-	77.6	64.9	61.5	40.5	49.4	41.1	49.2
Non-parent	50.4	58.3	52.5	51.5	54.1	64.0	49.7	55.2	53.3	63.2	64.5	48.0	43.5	38.8	39.7	30.3
*p* value	0.001				0.004				0.034				0.003			

**Table 4:** Factors Associated with Attitude Toward Grandparental Involvement.

	Beta (95% CI)	Beta (95% CI)	Beta (95% CI)
	Model 1	Model 2	Model 3
Wave ^a^			
2011	0.246 *** (0.208, 0.283)	0.249 *** (0.210, 0.287)	0.235 *** (0.193, 0.277)
2013	0.245 *** (0.208, 0.283)	0.241 *** (0.203, 0.279)	0.254 *** (0.213, 0.296)
2015	0.252 *** (0.214, 0.289)	0.253 *** (0.215, 0.292)	0.272 *** (0.230, 0.315)
Age ^b^	-		
15–24	-	0.086 ** (0.021, 0.150)	0.017 (−0.057, 0.092)
25–34	-	−0.015 (−0.075, 0.045)	−0.063 (−0.128, 0.003)
35–55	-	−0.071 *** (−0.110, −0.032)	−0.090 *** (−0.133, −0.048)
Male ^c^	-	0.034 * (0.004, 0.063)	0.051 ** (0.019, 0.084)
Education level ^d^	-		
Primary education or lower	-	−0.001 (−0.053, 0.052)	0.012 (−0.046, 0.070)
Secondary education	-	−0.010 (−0.052, 0.033)	0.003 (−0.042, 0.048)
Economically active ^e^	-	0.027 (−0.006, 0.060)	0.037 * (0.001, 0.073)
Marital status ^f^	-		
Married/cohabiting	-	−0.003 (−0.061, 0.055)	−0.063 (−0.131, 0.005)
Divorced/separated/widowed	-	−0.039 (−0.104, 0.027)	−0.056 (−0.138, 0.025)
Being a parent ^g^	-	0.126 *** (0.077, 0.174)	0.127 *** (0.070, 0.185)
New arrivals in HK ^h^	-	0.079 (−0.011, 0.168)	0.092 * (0.000, 0.185)
Satisfaction with relationship between family members and inter-generations	-	-	0.100 *** (0.069, 0.132)
Closeness to family member and inter-generations	-	-	0.001 (−0.030, 0.032)
Communications with family member and inter-generations	-	-	0.034 ** (0.012, 0.056)
Satisfaction with family life	-	-	0.041 *** (0.017, 0.065)
N (Sample size)	8802	8561	7088
R^2^	0.030	0.039	0.056
F-test	91.820 ***	24.999 ***	23.493 ***

Note. * *p* < 0.05, ** *p* < 0.01, *** *p* < 0.001. ^a^ Reference group = 2017 wave. ^b^ Reference group = 55 or above. ^c^ Reference group = female. ^d^ Reference group = post-secondary education or above. ^e^ Reference group = economically inactive. ^f^ Reference group = never married. ^g^ Reference group = do not have children. ^h^ Reference group = not new arrivals.

## Data Availability

Data available on request from the authors.
